# Remineralization of Demineralized Enamel and Dentine Using 3 Dentifrices—An InVitro Study

**DOI:** 10.3390/dj7030091

**Published:** 2019-09-02

**Authors:** Manjit Talwar, Ali Borzabadi-Farahani, Edward Lynch, Peter Borsboom, Jan Ruben

**Affiliations:** 1 Oral Health Centre Government Medical College & Hospital, Sector 32, Chandigarh 160047, India; 2 Orthodontics, Department of Clinical Sciences and Translational Medicine, University of Rome Tor Vergata, 00183 Rome, Italy; 3 Finchley Orthodontics, North Finchley, London N12 9EN, UK; 4 Biomedical and Clinical Research, School of Dental Medicine, University of Nevada (UNLV), 1001 Shadow Lane, Las Vegas, NV 89106-4124, USA; 5 Department of Oral and Maxillofacial Surgery, University Medical Centre Groningen, Hanzeplein 1, 9713 GZ Groningen, The Netherlands; 6 Department of Dentistry, Preventive and Restorative Dentistry, Radboud University Medical Center, Philips van Leijdenlaan 25, 6525 EX Nijmegen, The Netherlands

**Keywords:** demineralization, enamel, white lesions, remineralization

## Abstract

*Objectives:* To monitor the electrical resistance of artificially demineralized enamel and root dentine after exposure to different fluoridated dentifrices and, using transversal microradiography, to quantify remineralization. *Materials and methods:* This in-vitro blind investigation used 20 extracted teeth (four groups of five each). Each group was exposed to one test dentifrice [Colgate PreviDent (5000 ppm F), Colgate Winterfresh gel (1100 ppm F), Fluocaril Bi-Fluoré (2500 ppm F) and placebo (without fluoride)] three times daily for three minutes for 4 weeks. In between exposure to the test dentifrices, teeth were stored in a saliva storage solution. An Electrical Caries Monitor measured the electrical resistance at baseline and during the four-week test period at weekly intervals. The measurements were log transformed and Duncan’s multiple range test applied. Remineralization was quantified using transversal microradiography. *Results:* Log mean (SD) electronic carries monitor (ECM) measurements in enamel at baseline and after 4 weeks of exposure to the test dentifrices were 4.07(1.53) and 3.87(0.90) (Placebo-Fluocaril), 4.11(1.86) and 4.64(1.43) (Colgate Winterfresh gel), 4.81(0.9) and 4.21(1.20) (Fluocaril Bi-Fluoré), and 4.60(0.88) and 3.76(0.9) (Colgate PreviDent). Corresponding measurements in dentine were 2.13(0.89) and 3.06(0.87) (Placebo-Fluocaril), 1.87(0.63) and 2.88(1.32) (Colgate Winterfresh gel), 2.47(1.20) and 1.65(0.60) (Fluocaril), and 2.16(0.00), and 2.34(1.07) for Colgate PreviDent. Lesion depth (µm) after microradiography in enamel was 100.1 (Placebo), 50.6 (Colgate Winterfresh gel), and 110.2 (Fluocaril, and 97.1 (Colgate PreviDent), and corresponding values in dentine were 169.7, 154.8, 183.7, and 153.5. The correlation of ECM and microradiographic parameters was negative (*p* < 0.05). *Conclusion:* Exposure of artificially demineralized enamel and root dentine to fluoridated dentifrices and saliva storage solution resulted in remineralization as follows: Colgate Winterfresh > Colgate PreviDent > Placebo-Fluocaril > Fluocaril Bi-Fluoré. Remineralization in teeth of the Placebo dentifrice group may be attributed to the presence of calcium and phosphate ions in the saliva storage solution.

## 1. Introduction

Topical fluorides have had an important impact on the decline and prevention [1,2,3,4,5,6,7] of coronal and root caries. Fluoride dentifrices are the most widely used topical fluorides. Widespread reduction of dental caries worldwide has been attributed to extensive use of fluoride dentifrice [8,9,10]. The average reduction in dental caries attributed to fluoride dentifrices is assumed to be about 20 per cent [11]. Since the advent of dentifrices, it has been the goal of dental research efforts to identify more effective formulations for increasing the availability of fluoride. Fluoride dentifrices are safe and effective anti-caries agents, containing fluoride concentration in the range of 1000–1450 ppm. Sodium fluoride dentifrices have been found to inhibit caries lesion formation by 21.4% compared to a placebo dentifrice. Studies using sodium monofluorophosphate have shown caries inhibition of 22.5% [12]. High concentration fluoride dentifrices have been used for the management of patients with high risk of dental caries on prescription basis [13].

Remineralization occurs not only during periods of neutral pH, when minerals precipitate from the oral fluids in the enamel defects, but also during caries development. The role of fluoride in calcium phosphate precipitation is well documented. A pre-requisite for enamel lesion remineralization is the presence of partly demineralized crystallites, which act as nuclei for mineral deposition. For the formation of new crystals, a supersaturation greater than that of the oral fluids is necessary. Although lesion remineralization occurs through the regrowth of apatite crystals, physio-chemically the process is considerably more complicated. Enamel and dentine are different in structure and composition, which interferes in susceptibility to dental caries. The calcium hydroxyapatite [Ca_10_(PO_4_)_6_(OH)_2_] is the main mineral component of the tooth structure [14,15]. Enamel is formed of inorganic matrix (96%, *w*/*w*) and organic components such as proteins and lipids, and some water (4%, *w*/*w*). Mature dentine however, is about 70% mineral, 20% organic matrix, and 10% water by weight. Adding trace elements into the crystalline structure can modify the physico-chemical, mechanical properties, and the solubility of hydroxyapatite crystals. For instance, tooth hardness can be improved by adding zinc, or magnesium makes the substrata more porous, and fluoride makes the tooth structure more resistant to acid attack [15,16,17,18].

Dentine exhibits higher permeability to acid and a larger surface area of small crystallites, compared with that of enamel, contributing to enhanced rate of dissolution taking place in dentine [19]. The efficacy of fluoride dentifrices in the prevention and repair of enamel caries cannot be directly extrapolated to dentine. Remineralization will depend on the remaining mineral in the organic matrix as well as the enamel and dentine ultrastructure.

The evidence to support the prescription of high-concentration fluoride toothpaste, and particularly 5000 ppm toothpaste, to prevent caries is limited [20,21,22]. The most recent Cochrane review of different doses of fluoride toothpaste did not include any randomised controlled trials of 5000 ppm fluoride; however, it reported a dose-response relationship between the concentration of fluoride in toothpaste and caries prevention, with greater caries prevention for higher doses of fluoride [22]. According to this review, only a minority of studies assessed the adverse effects of toothpaste, and, when reported, effects such as soft tissue damage and tooth staining were minimal [22]. Another review of high-concentration fluoride toothpastes included four studies (randomised and non-randomised) with 5000 ppm fluoride toothpaste [23]. This review recommends further research on the use of population-based interventions using high-concentration fluoride toothpastes, and highlights that the evidence for dentifrices containing more than 2900 ppm is weaker than that for those containing 2800 ppm or less [23].

The quantitative level of fluoride through dentifrice required for the remineralization of enamel and dentine still needs to be established. The present investigation was undertaken to assess the effect of fluoridated dentifrices with different fluoride salts and concentrations on enamel and dentine. The objectives of the investigation were

1. To monitor electrical resistance in demineralized enamel and dentine using ECM after exposure to fluoridated dentifrices;
2. Using microradiography, to quantify mineral loss and the response of demineralized enamel and dentine to fluoridated dentifrices;
3. To assess the association between electrical resistance measurements of treated enamel and dentine (with placebo and fluoride dentifrices and saliva storage solution) with microradiographic data.

## 2. Materials and Methods

**Figure 1:**
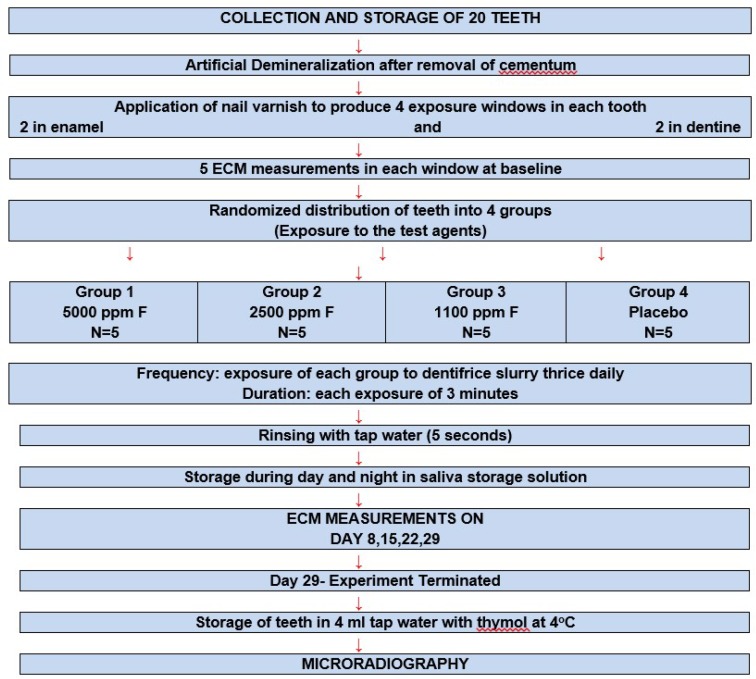
For testing fluoride dentifrice remineralization.

The investigation was blind, as all the dentifrices were coded and carried out in-vitro. The outline of the experiment is shown in Figure 1.

### 2.1. Collection and Storage of Teeth

Twenty sound (based on WHO 1997 criteria without any dental caries) molar teeth with complete root ends, extracted due to periodontal disease from subjects aged above 55 years, were collected. The collected teeth were stored for 10 days at 4 °C, in tap water with the addition of thymol crystals dissolved in 4 mL of alcohol to prevent growth of bacteria. All soft tissue was removed from the teeth prior to storage.

### 2.2. Artificial Demineralization

All surfaces of the teeth were cleaned with pumice slurry, and the cementum was removed using a rubber cup at slow speed (Procedure SFDD1001, SFDD2002, Materia Technica University, Groningen, The Netherlands). Following pumicing, the teeth were rinsed for 10 min in tap water and dried using tissue paper for three minutes at room temperature. The teeth were demineralized in a 6 wt% carboxymethyl cellulose gel (CMC, AKZO, The Netherlands) at pH 5.0 and 37 °C for 3 weeks. The gel contained 0.1 M lactic acid titrated to pH 5.0 with a 10 M KOH solution. The gel volume was 10 mL per sample. After in-vitro demineralization, all samples were washed carefully under running tap water for 30 min and rinsed again in distilled water for 30 s. After demineralization of the teeth, a red non-metallic nail vanish was used to produce exposure windows, two on the enamel surface at a distance >4 mm from the crown-root junction and two on the root (4 mm below the crown root junction). Depending on the size (diameter) of the tooth, four open windows of (4 × 12 mm) width were made using the same nail varnish. The remaining surface of the crown and root of all the teeth were also covered with the nail vanish. After the nail varnish dried, the teeth were stored in tap water with thymol.

### 2.3. Exposure to the Test Dentifrices

The twenty teeth were randomly distributed into four experimental groups of five each, as follows, and got exposed to the following dentifrices for 28 days, three times a day for 3 min:

G1, Colgate PreviDent 5000 plus (5000 ppm Fluoride Colgate Oral Pharmaceuticals Inc. MA02021 USA);

G2, Fluocaril Bi-Fluoré (2500 ppm Fluoride Creation des Laboratories Pharmaceutiques Goupil, France);

G3, Colgate Winterfresh gel (1100 ppm fluoride Colgate Palmolive Company, New York, 1022 USA); and 

G4, Placebo Fluocaril 250 code: 3ZJH01190), which had the same composition as Fluocaril Bi-Fluore 250 but did not contain fluoride.

In the control group (placebo dentifrice), the handling and storage conditions were similar to the other experimental groups. Between exposures to the test dentifrices, replacing tooth brushing, the teeth were stored in a saliva storage solution (with neutral pH) that contained potassium chloride (KCl 55.9 g), calcium chloride (CaCl_2_·2H_2_O 1.1 g), sodium cacodylate (16 g), and sodium hydrophosphate (KH_2_PO_4_ 5.5 g).

### 2.4. Remineralization

The teeth were removed from the storage container and rinsed in tap water for 10 min. For exposure to the test dentifrices, i.e., soaking (replacing tooth brushing), individual transparent 5 mL containers with caps were used. The test agents were three fluoridated and one placebo dentifrice. All the test dentifrices were coded.

At the termination of the experiment, the dentifrices were decoded. Exposure to the test dentifrices (soaking) was carried out three times daily, for 28 days, with each exposure of 3 min duration viz at 900 h, 1300 h, and 2100 h replacing the tooth brushing frequency. As the teeth had been distributed into four groups, dentifrice treatment in each group was carried out for each individual tooth. The container was filled with fresh 2 mL tap water, and freshly dispensed 1 mg toothpaste was added to make total of 3 mL. The procedure of adding toothpaste and tap water to the container was carried out quickly using disposable 5 mL syringes. The teeth were put in the container subsequent to the addition of tap water and dentifrice. The containers were tightly capped and shaken by hand for 3 min. During daytime, in between the dentifrice treatments, the teeth were stored in another 5 mL container, containing 5 mL fresh saliva storage solution (solution with a specific calcium and phosphate concentration without any fluoride, at room temperature). For storage at night, each individual tooth was wrapped in tissue paper wetted with fresh saliva-replacing solution. The teeth were completely covered by placing in a 5mL container that was capped and kept upright. The experiment was stopped on day 28.

### 2.5. Measurement of Tooth Resistance Using ECM

Electronic caries monitor (ECM, Lode Diagnostics BV, Groningen, The Netherlands) was used to measure the resistance in enamel and dentine following exposure to the test dentifrices, after 7 days (day 8), after 14 days (day 15), after 21 days (day 22), and after 28 days (day 29). Measurements were carried out on five sites per window, of which two windows were in enamel and two in dentine. A total of 20 ECM measurements were recorded for each tooth. Following this, the teeth were stored in tap water at 4 °C for microradiography to be carried out. Electronic caries monitor (ECM) was used to measure electrical resistance (lesion porosity) of artificially demineralized enamel and dentine.

### 2.6. Operating Principle

The ECM monitors the electrical resistance behaviour of a site on the tooth during controlled drying. By drying the surface, the resistance is determined only by the tooth volume, avoiding electrical guiding of the surface liquid (saliva). Electrical changes in resistance are measured using an Ohmmeter. The validation undertaken by researchers [24,25,26] indicates that the ECM has high sensitivity and specificity for the detection of enamel and dentine caries. The tip of the measuring electrode is placed at the suspected spot, and the reference electrode is connected to the root end, using a special in-vitro electrode with a wetted cotton contact with either water or 0.9% NaCl. With the standard ECM-scale, the electronic caries monitor records the measurement when an electrical contact between the measuring and reference electrodes is achieved (0.5 s delay, monitoring for constant signal level). The start of the measurement, i.e., drying of the tooth, is indicated by an acoustic signal (one beep). The measuring time of the standard ECM-scale is 5 s in total. The airflow (standard = 5 L/min) is fixed using an adjusted pressure reducer in the unit. The end of the measuring cycle is indicated by a double acoustic signal (double beep). ECM measurements were recorded at five sites (i.e., north, south, east, west, and centre) in each window in order to take care of any variation that occurred in different areas of the same window. At baseline, five ECM measurements were recorded at each of the five sites of the window, i.e., a total of 25 measurements in each window.

### 2.7. Microradiography

After remineralisation, three thin sections were cut of each sample with a rotating saw blade. The cutting direction was always perpendicular to the longest dimension of the window. The sections (80 µm thickness for enamel and 130 µm thickness for dentine) were placed in the sample holder of the microradiography camera; they were always wet to avoid shrinkage effects [27]. Microradiography was done as described in detail [28,29], and the lesion depth (Ld in µm) and the mineral loss in kg/m^2^, and in Vol%.µm (∆Z) were assessed. Microradiography was carried out by J Ruben in the Netherlands.

### 2.8. Repeatability of the Experiment (ECM Measurements)

In order to have valid results and test the examiner reproducibility, i.e., intra-examiner variability, at least twenty ECM measurements were recorded both in enamel and dentine; the measurements were found to be repeatable after 15 recordings. Repeatability was carried out on five premolar teeth. The co-efficient of variance was 5–8%, indicating that examiner reproducibility was high.

### 2.9. Statistical Analysis

The electrical resistance measurements’ mean integrated value in mega Ohms and mean end value in mega Ohms were log transformed, and Duncan’s multiple range test was applied. To assess the association between ECM measurements and microradiography data, the Pearson’s correlation test was used. Log ten transformation of integrated and end values was carried out, as the data was not normally distributed for the application of parametric tests.

## 3. Results

**Figure 2:**
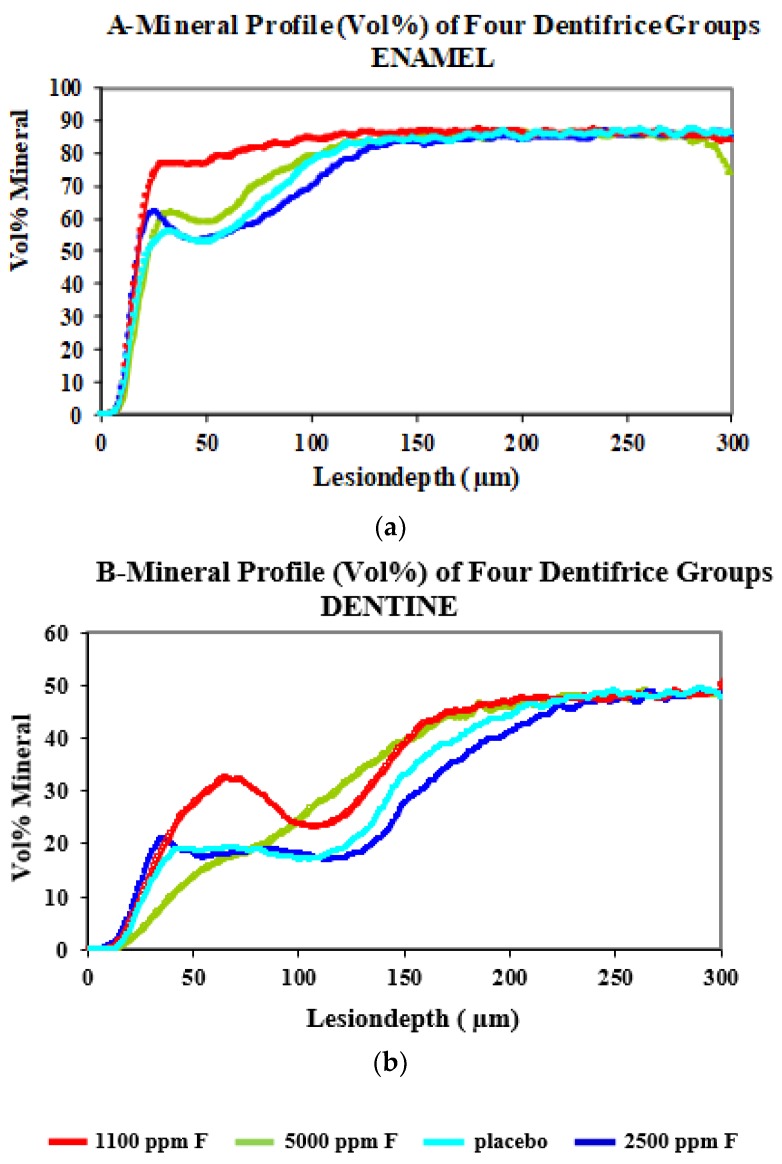
Mineral profiles (Vol%) of four dentifrices for enamel (**a**) and dentine (**b**). The X-axis shows the depth (micron meters).

In all groups, the mean electrical resistance measurements and standard error of mean were recorded as integrated (in Mega Ohms) and end values (in Mega Ohms). Peak electrical resistance measurements were recorded during the experiment (Table 1, Figure 2a,b). After seven days exposure of the teeth to the test dentifrices (thrice daily for 3 min and saliva storage solution), all fluoride dentifrices showed a peak in electrical resistance on day 8 followed by a slow decline till day 29 (after four weeks). This finding was similar for enamel and dentine and was statistically significant (*p* < 0.01). In the placebo dentifrice group in enamel, not much change was observed between day 8 and day 29. However, in dentine lower electrical, resistance was noted on day 8, which was statistically significant (*p* < 0.05), but increased slowly over a period of four weeks. After 28 days, a statistically significant increase in resistance was evident compared to baseline (*p* < 0.05). The day 29 ECM measurements remained elevated above baseline in enamel and dentine for the Colgate Winterfresh gel (1100 ppm F). However, for the Fluocaril Bi-Fluoré (2500 ppm F) the ECM measurements were lower than baseline on day 29 for enamel and dentine, and a similar trend was seen for the Colgate PreviDent dentifrice (5000 ppm F). Log10 transformation of integrated and end values was done because the data was not normally distributed, and for the application of parametric tests. Though the electrical resistance measurements showed an increase above baseline, these differences were not statistically significant on day 8, compared to baseline for enamel, but were statistically significant for dentine (*p* < 0.01) using the *t*-test. For the Colgate PreviDent dentifrice group, the electrical resistance measurements on day 8 were statistically higher in enamel and dentine, compared to baseline (*p* < 0.01). 

Duncan’s multiple range test was applied, and the ECM measurements in enamel and dentine on day 8 showed the following trend: Colgate Winterfresh gel > Fluocaril Bi-Fluoré > Colgate PreviDent > Placebo Fluocaril (*p* < 0.01). On day 15, Colgate PreviDent showed the highest electrical resistance values in enamel and dentine compared to other dentifrices.

Lesion depth and mineral loss were assessed using microradiography in enamel and dentine (Table 2 and Table 3). Lesion depth measured in micron meters was the least with Colgate Winterfresh gel > Colgate PreviDent > Placebo (Fluocaril) > Fluocaril Bi-Fluoré. Mineral loss measured in Kg/m^2^ indicated a similar trend. R is the ratio of mineral loss value/lesion depth (Z is vol%. µm). A similar trend was seen for dentine. At a depth of 100–150 µ, Colgate PreviDent performed better than Placebo and Fluocaril Bi-Fluoré. At a lesion depth of 150 µ, its effect was comparable to Colgate Winterfresh gel. Association of the ECM measurements (day 29) with microradiographic data was assessed using the Pearson’s correlation test (Table 4 and Table 5). Findings showed that as the electrical resistance increased the lesion depth and mineral loss values decreased. ECM and microradiographic data were negatively correlated.

## 4. Discussion

The ECM allows measurements to be made in the range of 1 Kilo Ohm to >10 Giga Ohm. The electrical resistance value of a tooth depends on the local porosity of the measured tooth site, the amount of liquid in the porous area, temperature, mobility of liquid in the porous area, and ion concentration of the liquid. To avoid the influence of any surface liquid, the tooth surface was dried using controlled airflow drying procedure. To have reproducible reading, ten ECM measurements were recorded (five in each window, two in enamel, and two in dentine). Rationale for exposing the teeth to the test dentifrices thrice daily was that tooth brushing frequency (in-vivo) for the prevention of root and coronal caries is three times daily. Following exposure to the test dentifrices, the teeth were placed in plastic vials containing saliva storage solution (with neutral pH) in an attempt to simulate the in-vivo conditions. During the night, the teeth were wrapped in tissue paper wetted with the saliva storage solution simulating the reduced salivary flow rate at night. According to Kusano et al., the use of fluoride dentifrice at night to remineralize daily mineral loss maybe preferable to brushing in the morning to inhibit the demineralizing episodes of the day [30].

ECM measurements were recorded after 7, 14, 21, and 28 days of exposure to the test dentifrices and saliva storage solution. In the study of Dunipace et al., extending the experimental period from 7 to 14 days resulted in a dramatic effect on dentine fluoride uptake [31]. In an experiment by Von Der Fehr et al., lesions remineralized and disappeared in three weeks [32]. In view of this, the experimental period of four weeks in this investigation was appropriate. The microradiography is the most practical technique for direct and quantitative measurement of mineral content, mineral changes, and mineral distribution [33]. Water contains several ions that can affect de- and re-mineralization phenomenon. Water used in the present experiment was deionized. Electrical measurements for enamel and dentine over a four-week period were different. 

Dentine remineralization is more challenging than enamel remineralization due to the higher percentages of the organic matrix in dentine (20%) [34]. Apparently, spontaneous precipitation or nucleation of mineral on the organic matrix (mainly type I collagen) does not contribute much to the dentine remineralization, and the growth of residual crystals in the lesions plays a major part in the process [34,35,36]. Remineralization of the lesions extending into the dentine with fluoride [34,37,38] is possible, but; this process depends on the amount of residual crystals and supplement of calcium and phosphate ions [34,39], which takes longer time (compared to enamel).

Dentine demineralizes more because of the proteolytic breakdown of the collagenous matrix, which form a higher percentage compared to enamel [34,35,36,37,38,39,40]. Lesions in dentine are of a greater depth [41]. Dentine has a higher organic matrix, is more porous, and may permit caries to progress more rapidly in dentine than enamel [42,43]. ECM measures tooth resistance (lesion porosity), and microradiography quantifies remineralization.

Compared to the Placebo dentifrice, all fluoridated dentifrices had higher tooth resistance values in enamel and dentine. In this model system, the best results were observed for Colgate Winterfresh gel > Colgate PreviDent > Fluocaril Bi-Fluoré > Placebo Bi-Fluore. Higher fluoride dentifrices did not provide superior remineralization to 1100 ppm fluoride dentifrice both in artificially demineralized enamel and dentine. The Fluocaril dentifrice contained two fluoride salts, i.e., sodium fluoride and sodium monofluorophosphate, and the combination did not show any superiority in terms of remineralization. This was probably because the fluoride in monofluorophosphate was not hydrolyzed, as pH cycling was not used and available for remineralization. Based on the findings of this study, it may be concluded that high-fluoride agents result in a choking-off phenomenon, where uptake of fluoride, calcium, and phosphate ions is prevented. Low but constant level of fluoride may offer a greater therapeutic benefit by preventing demineralization and bringing about remineralization than single application of high-concentration topical fluorides. However, in patients with xerostomia, Sjogren’s syndrome, and radiation caries, and older adults who are home-bound due to impaired physical and mental disabilities and have difficulty in carrying their daily oral hygiene regimen, brushing once daily with a high-fluoride dentifrice would be a more pragmatic approach. The findings may also have implications for the management of white spots in high-risk orthodontic patients [44,45]. Colgate PreviDent 5000 plus dentifrice showed superior remineralization in dentine at a depth of 100–150 µ, possibly because the remineralizing potential is greater in a tissue that demineralizes more.

## 5. Conclusions

Exposure of artificially demineralized enamel and root dentine to fluoridated dentifrices and saliva storage solution resulted in remineralization in the following order: Colgate winterfresh > Colgate PreviDent > Placebo-Fluocaril > Fluocaril Bi-Fluoré. Remineralization in teeth of the Placebo dentifrice group may be attributed to the presence of calcium and phosphate ions in the saliva storage solution.

## Figures and Tables

**Table 1:** Peak electrical resistance of enamel and dentine during the experiment.

Test Dentifrices	Tissue Enamel					Tissue Dentine				
	DAYS OF EXPERIMENT									
	0	8	15	22	29	0	8	15	22	29
Placebo Log10 Int value.	4.07 ± 1.53	3.92 ± 1.16	4.06 ± 1.16	3.56 ± 1.18	3.87 ± 0.90	2.13 ± 0.89	1.82 ± 0.98	2.15 ± 0.88	2.60 ± 0.60	3.06 ± 0.87
Log10 End value.	1.28 ± 0.62	1.33 ± 0.30	1.37 ± 0.27	1.23 ± 0.28	1.33 ± 0.23	0.69 ± 0.34	0.47 ± 0.63	0.71 ± 0.34	0.93 ± 0.24	1.07 ± 0.31
Colgate Winter Fresh gel Log10 Int value.	4.11 ± 1.86	5.79 ± 1.21	5.01 ± 1.15	4.64 ± 1.48	4.64 ± 1.43	1.87 ± 0.63	4.00 ± 2.02	2.19 ± 1.90	2.64 ± 1.73	2.88 ± 1.32
Log10 End value.	1.24 ± 0.69	1.73 ± 0.24	1.59 ± 0.23	1.47 ± 0.40	1.49 ± 0.32	0.56 ± 0.39	1.20 ± 0.72	0.44 ± 0.97	0.78 ± 0.63	0.98 ± 0.41
Fluocaril **Bi-Fluoré** Log10 Int value.	4.81 ± 0.9	5.26 ± 1.72	3.82 ± 1.40	4.17 ± 1.31	4.21 ± 1.20	2.47 ± 1.20	3.87 ± 2.31	1.33 ± 0.72	2.00 ± 0.79	1.65 ± 0.60
Log10 End value.	1.55 ± 0.20	1.59 ± 0.42	1.29 ± 0.32	1.39 ± 0.31	1.40 ± 0.29	0.80 ± 0.50	1.00 ± 1.09	0.10 ± 0.79	0.53 ± 0.66	0.40 ± 0.57
Colgate Prevident Log10 Int value.	4.60 ± 0.88	5.50 ± 0.76	5.16 ± 1.40	4.02 ± 1.20	3.76 ± 0.90	2.16 ± 0.00	4.93 ± 0.55	2.92 ± 1.49	2.50 ± 1.20	2.34 ± 1.07
Log10 End value.	1.50 ± 0.20	1.70 ± 0.14	1.60 ± 1.00	1.34 ± 0.35	1.30 ± 0.24	0.73 ± 0.37	1.59 ± 0.11	0.93 ± 0.60	0.82 ± 0.42	0.76 ± 0.42

**Table 2:** Transversal microradiography data for the enamel.

Test Dentifrice’s	Lesion Depth (Ld, µm) Mean ± SD	Mineral Loss (Kg/m^2^) Mean ± SD	Mineral Loss Value (∆Z) Vol%. µm	Ratio of Mineral Loss ∆Z/Ld
Colgate PreviDent (5000 ppm F)	97.1 ± 25.7	0.10 ± 0.02	3062	32.8
Fluocaril (2500 ppm F)	110.2 ± 35.4	0.11 ± 0.05	3619	33.5
Colgate Winterfresh Gel (1100 ppm F)	50.6 ± 35.8	0.05 ± 0.02	1613	41
Placebo (Non-Fluoridated)	100.1 ± 20.3	0.11 ± 0.02	3414	34.4

**Table 3:** Transversal microradiography data of dentine.

Test Dentifrice’s	Lesion Depth (Ld, µm) Mean ± SD	Mineral Loss (Kg/m^2^) Mean ± SD	Mineral Loss Value (∆Z) Vol%. µm	Ratio of Mineral Loss ∆Z/Ld
Colgate PreviDent (5000 ppm F)	153.5 ± 22.2	0.14 ± 0.03	4420	28.7
Fluocaril (2500 ppm F)	183.7 ± 32.2	0.16 ± 0.03	5152	28.1
Colgate Winterfresh Gel (1100 ppm F)	154.8 ± 22.6	0.12 ± 0.03	3696	23.7
Placebo (Non-Fluoridated)	169.7 ± 25.9	0.15 ± 0.03	4867	28.5

**Table 4:** Association of electronic carries monitor (ECM) with microradiography data of enamel (Pearson’s correlation test).

Variables	Lesion Depth	Minerals Loss Value	Volume of Mineral Loss	ECM
Lesion	1.00	0.99	0.99	−0.73
Mineral	0.99	1.00	1.00	−0.72
Volume	0.99	1.00	1.00	−0.72
ECM	−0.73	−0.72	−0.72	1.00

**Table 5:** Correlation of ECM with microradiography data of dentine (Pearson’s Correlation test).

Variables	Lesion Depth	Mineral Loss	Volume of Mineral Loss	ECM
Lesion	1.00	0.85	0.85	−0.54
Mineral	0.85	1.00	1.00	−0.49
Volume	0.85	1.00	1.00	−0.49
ECM	−0.54	−0.49	−0.49	1.00
